# Motor Development Scales Validated in Spanish Populations of Children Aged 0–18 Months: Systematic Review

**DOI:** 10.3390/children12091106

**Published:** 2025-08-22

**Authors:** Elena Cala de la Torre, Elena Pinero-Pinto

**Affiliations:** 1 Physiotherapy Department, Faculty of Nursery, Physiotherapy and Podiatry, University of Seville, 41009 Seville, Spain; epinero@us.es; 2 Instituto de Biomedicina de Sevilla-IBiS (Hospitales Universitarios Virgen del Rocío y Macarena/CSIC/Universidad de Sevilla), 41013 Seville, Spain

**Keywords:** motor development, motor skills, evaluation tools, child, validation

## Abstract

**Background/Objectives**: The assessment of motor development in early ages is considered fundamental for the early identification of alarm signs that indicate the possibility of a child suffering from a developmental disorder. To this end, it is necessary to use reliable evaluation tools that have been specifically validated in the target population. The main aim of the current review was to examine the existing motor development assessment tools validated in Spanish populations of children aged 0–18 months. **Methods**: A systematic review was carried out following the PRISMA (Preferred Reporting Items for Systematic reviews and Meta-Analyses) declaration criteria, and it was registered in PROSPERO. A bibliographic search was conducted in the PubMed, Scopus, Web of Science, and Cochrane databases, using terms related to motor development, assessment tools, and validation. The COSMIN verification list was employed to evaluate the quality of the included scales, and the QUADAS-2 instrument was used to analyse the quality of the included studies. **Results**: A total of 7 studies were included in the review, all of which were validation studies of motor development assessment tools in Spanish populations of children aged 0–18 months. Moreover, they all show heterogeneity with respect to their characteristics, such as target population, sample size, and metric properties. **Conclusions**: The present systematic review provides relevant information about the characteristics and methodological quality of motor development assessment tools validated in Spanish populations. There are currently very few of these evaluation tools, as well as limitations in regard to theories that support them, their metric properties, and the methodological quality of their validation studies. Therefore, we confirm the need for validating updated motor development tools to improve the detection, prognosis, and evaluation of children with developmental disorders or at risk of suffering from them.

## 1. Introduction

Motor development is a process of change in which movement patterns and motor skills are acquired [1]. This change in motor behaviour begins in childhood, when there is a drastic increase in motor skills [2,3]. To study motor development, changes in early life and ageing are analysed, although there are still limitations in our understanding of this [4]. The definition of “early development” is used to describe the changes that occur from birth to young adulthood, and “late development” to describe it from young adulthood to old age [5].

The theories that explain the concepts about motor behaviour have changed throughout time [2]. Firstly, the earliest theories considered motor development as a hierarchical and invariable sequence, an innate and maturing process [6] where changes were caused by the evolution of reflexes and depended on the control of higher orders of the central nervous system (CNS) [7]. For this reason, it is necessary to identify assessment tools that identify changes in motor development in the first months of life, due to the maturation process itself [8,9].

It has been recently reported that motor behaviour is not organised through reflexes, but it is based on spontaneous activity and begins to be modulated from the foetal age, as a result of the continuous interaction of neural networks that mediate a motor action [3]. Thus, the two current theoretical frameworks that explain motor development are Dynamic Systems Theory (DST) [10] and Theory of Neuronal Group Selection (TNGS) [2]. Both theories state that motor development is a non-linear process with multifactorial origin and transition phases, and they both recognise the importance of experience and context. TNGS adds the relevance of genetic information and epigenetic cascades.

The evaluation of motor development at early ages is essential for the early detection of motor delays, variations, or disorders, which allows for initiating early intervention following the principles of neuroplasticity [11,12]. Children aged 0–18 months have specific developmental characteristics that are not comparable or measurable with larges stages of development [2].

Several studies suggest that intervention during childhood is more effective in periods of high brain plasticity [13]. The first 12 months of life pose a critical period in brain development, since the myelination process increases [11]. A recent study has shown that the human brain retains its capacity for change throughout life, although it is less so in adulthood than in childhood. There is a remarkable capacity to reorganise neural circuits even at age 60, so research is currently underway to optimise motor function throughout life [14].

It is difficult to establish a clear diagnosis of motor disorder before the age of 18 months, as this period is characterised by maximum neural plasticity and adaptive behaviour has not been reached yet, although risk signs can be identified [2,11,12,15]. Depending on child age, signs, and motor areas to be evaluated, professionals employ valuations that contemplate the attainment of motor milestones, movement quality, reflexes, muscle tone, and neuromotor or postural reactions [16]. The choice of a specific valuation tool must be conducted according to the following evaluation criteria or objectives: discrimination, prediction, or longitudinal evaluation [16,17]. Moreover, other studies have also demonstrated the importance of applying reliable, sensitive, and specific scales that have been validated in the target population [17].

The main aim of the current study was to examine the existing motor development evaluation tools validated in Spanish populations aged 0–18 months. Once identified, it was necessary to analyse the psychometric properties of these scales and compare their characteristics and scopes of application, in order to determine their clinical and research usefulness. This review is justified by the importance of knowing the psychometric properties of motor evaluation tools and their validation in Spanish populations, with the aim of ensuring that their selection and use are adequate. Furthermore, it may contribute to the scientific literature as it provides solid knowledge on the applicability of tools in the clinical and research practice, serving as a reference for professionals.

## 2. Materials and Methods

A systematic review was carried out following the PRISMA (Preferred Reporting Items for Systematic reviews and Meta-Analyses) declaration criteria [18]. The review was registered in the PROSPERO database (identification number: CRD420250644451).

### 2.1. Search Strategy

A bibliographic search was performed in the following databases: PubMed, Web of Science (WoS), Scopus, and Cochrane.

The search terms employed were combined with Boolean descriptors OR and AND. In general terms, the search equation used was as follows: (assessment OR tool OR evaluation OR examination OR test OR scale) AND (“motor skill” OR “motor development” OR neuromotor OR motor OR neurological OR psychomotor) AND (spain OR spanish) AND (validation) AND (infant OR child).

### 2.2. Selection Criteria

The inclusion criteria of this review for the selection of studies were as follows: [1] scales that measured child motor development; [2] scales in which the evaluation age range was 0–18 months; [3] scales that were validated in Spanish populations; [4] populations with neurotypical development, developmental disorder, or psychomotor delay; [5] full-term or preterm children, with the evaluation considering their corrected age. The exclusion criteria were as follows: [1] scales developed or validated in international contexts without adaptations to the Spanish population; [2] letters to editors, summaries, or conference minutes.

### 2.3. Selection Process

The general article selection process was conducted using the latest version of Rayyan application from 2025 (Rayyan—AI Powered Tool for Systematic Literature Reviews) [19]. After discarding duplicates, the articles were independently selected by the two main researchers based on title and abstract. To increase confidence in the article selection, all potentially relevant articles were reviewed by both researchers together. After reading all studies, two principal research reached a consensus to establish which articles fulfilled the inclusion criteria. Any disagreement was solved by a third researcher.

Once the articles selected by title and abstract were obtained, a full-text reading of the articles was carried out to verify which of them met the inclusion criteria by the first author. The second author then reviewed the full text of the included articles and agreed.

The process led to the selection of 7 articles that met the inclusion criteria to be systematically reviewed.

### 2.4. Data Synthesis

The data were extracted by the main researcher and verified by a second researcher. A protocol was established between the two principal researchers. Disagreements between both reviewers were resolved by a third reviewer, who assessed the information independently to resolve discrepancies.

A summary table of the included motor development assessment tools was designed, which gathers the author/s, year of publication, country of origin, scale description, population, age range, application instructions, and psychometric properties.

A second table was also included with the description of the items evaluated by the assessment tools included in this review: gross motor development, reflexes, postural control, balance, coordination, locomotion, object handling, grip, muscle tone, asymmetries, quality, fluidity, and social behaviour (response to stimuli).

### 2.5. Article Quality Evaluation

The instrument used to evaluate the quality of the identified scales was COSMIN (COnsensus-based Standards for the selection of health Measurement Instruments). This tool is a verification list that assesses the metric properties of studies included in reviews. It consists of a 4-point scale, which classifies each evaluation into four levels (“very good”, “adequate”, “doubtful”, and “inadequate”), and 9 sections, which correspond to 9 psychometric properties with each section containing 5–18 items related to study design and statistical methods. COSMIN does not consider the psychometric properties that have not been addressed in the articles [20].

To evaluate the methodological quality of the articles that validated the scales, QUADAS-2 (Quality Assessment of Diagnostic Accuracy Studies) was employed. This scale analyses studies of diagnostic criteria validation, and it was used to assess the risk of bias (“low”, “high”, or “uncertain”) and applicability of the scales included in the reviewed studies through the questions that make up its seven items [21].

A table was created by the first author with the items to be qualitatively assessed. It was completed independently by the two researchers, and discrepancies were resolved by a third reviewer.

## 3. Results

**Figure 1:**
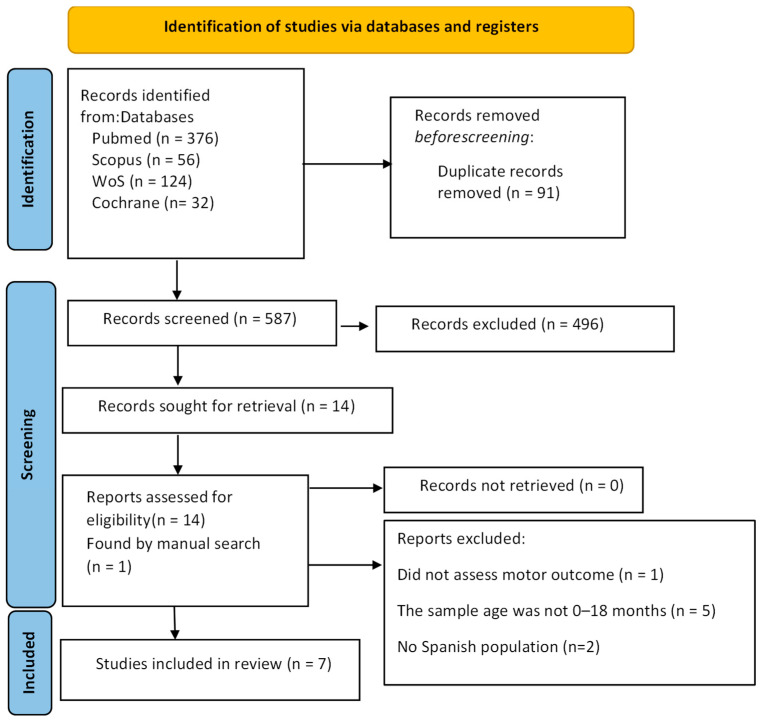
PRISMA 2020 flowchart.

The results of the article search and selection are presented in Figure 1. After discarding 91 duplicates, 587 articles were identified, of which 496 were excluded by title and abstract. A total of 14 articles were full-text read, of which 8 were excluded for not meeting the inclusion criteria. A doctoral thesis was identified through manual search on the Internet, which met the inclusion criteria, and was therefore incorporated into the current review. Lastly, a total of seven studies were included in this systematic review.

### 3.1. Characteristics of the Included Studies

Table 1 shows the descriptive data of the selected articles (author, year, country of origin, definitions, indications, instructions of use, and psychometric properties). Table 2 contains a check list of motor development variables that measure the evaluation tools.

With regard to the target population of the included scales, two of the latter evaluated children with motor development delay [22,23], four of them assessed preterm children [8,26,27,28], and one scale evaluated children with cerebral palsy (CP) [24,25]. However, all of them can be used in children with typical development to assess motor development variables. A total of five scales begin evaluating from birth [22,23,26,27], one of them from the age of 2 months [8] and one from the age of 5 months [24,25]. All included scales are administered through direct observation of the child, although three of them [8,26,28] require the therapist to perform changes in order to observe triggered responses. The administration time also varied among the evaluation tools, with Premie-Neuro [27] and HINE [8] being the ones that take the shortest time to administer, between 4 and 15 min, and the rest can take up to 20–30 min [22,28] and 45–60 min [23,24,25,26,27].

The motor development variables that were most addressed in the evaluation scales are milestones of gross motor development [8,22,23,24,25], reflexes [8,23,27,28], postural control [8,22,23,24,25,26,27,28], and response to stimuli [8,24,25,26].

With respect to the psychometric properties, most of the studies analysed interobserver reliability [23,27], intraobserver reliability [28], or both [8,22,24,25]. Five studies measured the internal consistency [22,23,26,27,28], one of them calculated the construct validity [27], and one of them determined the concurrent validity [22].

### 3.2. Study Quality Evaluation

The results of the QUADAS-2 instrument are presented in Table 3. Among the 49 sections of QUADAS-2 in this study (seven items for seven studies), 10 showed a low probability of risk of bias, 14 a moderate probability, and 3 a high probability. In regard with applicability, 11, 8, and 3 sections obtained a low, moderate, and high probability, respectively. Two studies presented greater uncertain probability of risk of bias and applicability [22,28], with the rest of the sections of the mentioned studies showing a low probability. The rest of the included studies contain sections of high probability: one section [23,24,25,27], and two sections [26]. Only two studies have four sections of low probability [8,23] and one of them did not even show a low probability [8].

Table 4 presents the methodological evaluation quality based on the COSMIN verification list.

Two sections (measurement error and hypothesis test) were removed, since they were not considered in any of the seven articles included. Among the seven sections used, transcultural adaptation was only addressed in one study [24,25]. In most of the sections of the verification list, the included studies obtained an “adequate” qualification. None of the studies presented a “very good” qualification, and only one study presented an “inadequate” qualification [26].

## 4. Discussion

One of the main findings of this systematic review is the heterogeneity in the target population of the motor development scales validated in Spanish populations. Among the identified scales, two of them evaluate children with motor development delay [22,23], four of them assess preterm children [8,26,27,28], and one of them evaluates children with CP [24,25]. This variability could indicate that there are scales validated for specific populations, which enables a more precise and sensitive evaluation in said populations and follow-up of development attending to the particularity of each scale. On the other hand, there are other populations that have not been studied, and thus there are no specific tools available to measure motor development in them.

The most current theory of motor development, TSGN, states that in the primary variability phase the spontaneous activity of the nervous system explores all available motor possibilities, increasing the variation in motor behaviour. This means that it helps fine-tune the genetically based structure of the somatosensory cortex. Spontaneous activity prepares the nervous system to adapt motor behaviour to the situation presented until the secondary variability phase begins, and it is at this moment that the nervous system uses afferent information to select the most appropriate behaviour for the situation. Therefore, TSGN maintains that the evaluation of the child should be carried out by observing the baby’s spontaneous motor behaviour in different specific situations [2,11].

All the evaluation scales included in this review are administered through direct observation of spontaneous movement. The fact of not using video recording can cause information to be lost when recording what was observed at the same time as administering the scale. Only two scales, in addition to observation, perform changes to evaluate responses to triggered movements [27,28], which are not supported by the current theory of motor development.

In relation to the items of motor development included in the scales, the only item in common for all scales is postural control. The second most addressed items are milestones of gross motor development [8,22,23,24,25], primitive reflexes [8,23,26,27,28], and response to stimuli [8,26,27,28]. The heterogeneity observed in the items of the scales is due to the fact that there are two groups of tools: one assesses motor development exclusively and the other includes neurological valuation. Therefore, the evaluation objective is not the same for all scales. The four scales that include items of social behaviour and response to stimuli [8,26,27,28] are also the ones that include neurological valuation. The object handling item is only included in one scale [23] and the grip item is included in two scales [8,23], which indicates that in the current evaluations of motor development no relevant milestones of fine motor skills are included. Mentioning the TSGN, by evaluating only one scale, object handling, we are losing information about the variability and adaptability of upper limb movements. Muscle tone [8,26,28] and asymmetries [8,27,28] are only measured in three scales, and these two items are both included in HINE and Premie-Neuro. No scale assesses the variation and adaptability that recent theories justify; through observation, the execution or non-execution of items presented is noted, without evaluating the quantity of movements (variation) and their adaptation to the situation (adaptability).

In the analysis of motor development, it is relevant to know the importance of identifying signs for the early detection of possible neurodevelopmental disorders, although it is necessary to consider that the high brain plasticity in childhood interferes with said detection [2,15,29]. It is known that prediction is most accurate when the brain structures have been replaced with permanent circuits. This takes place at the age of 3 months (the cortical subplate of the prefrontal and parietotemporal cortices, as well as the external granular layer of the cerebellum, have disappeared), thus a specific, accurate diagnosis cannot be established until this transition has occurred [3,15]. All scales included in the review begin their evaluation in the age range of 0–5 months; therefore, the obtained results may suggest a risk of motor alteration, and it is enough to initiate early intervention although a specific diagnosis cannot be established. Motor and neurological evaluations, along with global development and neuroimage evaluations, are considered to be the most relevant methods for early detection [15].

The motor evaluation tools that are most widely used in young children, according to a systematic review published in 2008 by Kirsten R. Heineman and Mijna Hadders-Algra, are as follows: Prechtl’s General Movements Assessment (GMA), Hammersmith Infant Neurological Examination (HINE), Test of Infant Motor Performance (TIMP), Alberta Infant Motor Scales (AIMS), Touwen infant neurological examination, Amiel-Tison neurological examination, Bayley’s Scales of Infant Development (BSID), Peabody developmental Motor Scales-2 (PDMS-2), Movement assessment of infants (MAI), Neuromotor behavioural inventor (NBI), Toddler and infant motor evaluation (TIME), Structured observation of motor performance (SOMP), Infant neurological international battery (Infanib), and Primitive Reflex Profile (PRP) [30]. The most popular of these scales is GMA [29], as well as the best clinical tool for the diagnosis of CP [15,29], which was not included in this systematic review since there are no validation studies in Spanish populations for said disorder. GMA consists of the evaluation of the CNS through direct observation of the infant’s spontaneous movements [31]. Of the seven evaluation tools analysed in this review, four of them are among the most widely used [8,22,23,28]

Making an international comparison, several international studies have validated motor development scales in infants and young children, and methodological and cultural differences have been found with respect to the Spanish context. The AIMS was developed and validated in Canada with a normative sample of more than 2000 infants, with high reliability (ICC > 0.98) and concurrent validity. The results in Spain regarding psychometric properties are similar, but the sample size is smaller [32]. Similar results were obtained in the original validation of the PDMS-2 in the US and Canada, where concurrent and criterion validity were also analysed, obtaining excellent correlations with other scales administered (AIMS and BSID-III) [33]. The HINE, Premie-Neuro, and TIMP scales have been validated internationally as early neurological assessment tools, especially the former for the detection of cerebral palsy in at-risk infants. All of these scales had better results in their original validation studies and used a larger sample size [9,34,35]. The differences in results obtained in each validation may suggest that motor development curves differ in different populations. International comparisons demonstrate the importance of validating motor development scales in specific contexts, as sociocultural differences can significantly affect the observed development patterns.

Furthermore, the outcome measure varies among the included scales. Two scales interpret the results attending to percentiles [22,23]. The percentiles represent values that indicate the percentage of the distribution that is equal to or lower than a particular score, with a mean value of 50. The difference between these two scales [22,23] is that AIMS [22] indicates motor alteration (under percentile 5), risk of motor alteration (between percentiles 5 and 10), and typical development (from percentile 10), whereas PMDS-2 [23] does not indicate specific values, showing only whether the score is far or close to the mean. Moreover, the latter scale includes more scores (scaled score and development quotient). Four scales [8,26,27,28] interpret the results based on the number of items marked and a total sum (total score), which is translated differently depending on the number of total points obtained.

With regard to the evaluation of the methodological quality with the COSMIN verification list, most of the results were classified as “adequate” or “doubtful”, and none were classified as “very good”. The “adequate” classification was obtained by three studies in internal consistency [22,23,28], four in reliability [22,23,24,25,28], five in content validity [8,22,24,25,26,28], four in structural validity [8,22,23,28], three in sensitivity [8,22,28], and one in transcultural validity [25]. The “doubtful” classification was presented by four studies in internal consistency [8,24,26,27], two in reliability [8,27], two in content validity [23,27], three in structural validity [24,26,27], and all in criterion validity [8,22,23,24,25,26,27,28]. This information suggests that there are studies with limitations and others with good methodological quality. Regarding the systematic reviews of motor development evaluation scales, only one study used the COSMIN verification list in its methodology [36], and most of them were classified as “doubtful” as they did not include missing data, had a small sample size, and employed inadequate statistical methods.

In regard with the sample size, the COSMIN verification list considers a sample of less than 30 participants as “inadequate”, 30–49 participants as “doubtful”, 50–99 participants as “adequate”, and over 100 participants as “very good”. Two studies [8,27] obtained a “doubtful” score in internal consistency, reliability, and criterion validity since they included a sample size that was considered deficient. Four studies [22,24,27,28] had a sample size of 50–99 participants, although they did not obtain a score above “adequate” for other reasons. A small sample size is considered a bias when extrapolating the results to the general population. The studies with the highest score [22,23,28] had an “adequate” sample size and presented good psychometric properties, with all of them showing an “excellent” score in Cronbach’s α to characterise the internal consistency strength.

In the comparison using QUADAS-2, there was a partial coincidence for the results obtained in methodological quality. In regard with the “reference test” section, only one study [22] used one of these tests to compare results, whereas the rest of the studies did not use any reference test; thus a bias can be inferred since the obtained results were not compared with a standard or previously validated test.

The validation of the motor development scales in specific populations should be a priority in research on early intervention, as there are currently few studies available in this respect. Children aged 0–18 months have specific developmental characteristics that are not comparable or measurable with those at later stages of development. Therefore, since existing ones for older children or adults cannot be adapted, existing ones specific to our language and population must be validated.

The articles included in this review show heterogeneity in terms of their characteristics, such as target population, sample size, and psychometric properties. The motor development evaluation scales are viable tools for the clinical practice, although few studies are conducted and there are few of these tools validated in Spanish populations. The present systematic review is presented as a necessary compilation of motor development assessment tools validated in Spanish populations. It contains the analysis of the characteristics and methodological quality of the scales validated in Spanish populations to date. This document will allow professionals to know the validated scales, compare the available tools, and identify the most adequate scale for each child.

This review is expected to allow developing evaluation tools that are based on current theories of motor development, as well as ensuring that future validation studies show a better methodological quality. Consequently, it will be possible to improve the quality of the evaluations of children and, therefore, the interventions.

## 5. Conclusions

The limitations of the present review begin with the small number of scientific studies found based on the selection criteria established. Currently, there are few motor evaluation tools validated in Spanish populations in the age range of 0–18 months. Moreover, the included scales measure different variables of motor development, which may pose a lack of homogeneity in terms of what is important to evaluate in motor development. This is a possibility as the scales are founded on different motor development theories and they also have different evaluation objectives. Another point to consider is the methodological quality of the validation studies. The sample size in several studies is small, and thus the results cannot be generalised, resulting in psychometric properties with low scores.

Furthermore, no publication date limit was set. This suggests that some validation studies may be old and were not subjected to the scientific requirements that are demanded by current prestigious journals.

To conclude, this systematic review ascertains the need to validate more current motor development evaluation scales in Spanish populations. The early detection of alarm signs that suggest the possibility of suffering from a neurodevelopmental disorder is important, and this requires available quality evaluation tools that are validated in specific populations. This would also pose improvements in the detection, prognosis, and evolution of children with neurodevelopmental disorders or at risk of suffering from them.

## Figures and Tables

**Table 1:** Characteristics of the included scales.

Scale, Author, Year	Country of Origin	Scale Description (Definition and Number Items)	Population	Age Range	Administration Time	Instructions of Use	Outcome Measure	Psychometric Properties	Proportion of Children Aged 0–18 Months Included in the Study Population
AIMS, Martha C. Piper and Johanna Darra, 1994 [22]	Canada	Observational scale (spontaneous motor skills). 58 items (21 PP, 9 SP, 12 Si, 16 St).	Children with TD, risk, motor delay.	0–18 months	10–30 min	Direct observation. Mark the motor window of the child and write down the observed items, giving a score of 1 point.	Percentile.	IC (α TS-0.99, P-0.97, Sp-0.88, St-0.97, S-0.96) Intra-R (ICCs 0.94–1.00 CI 95%) Inter-R (ICCs 0.95–1.00 CI 95%) CoV (0.973)	100%
HINE, Lilly Dubowitz, 1981 [8]	UK	Neurological evaluation. 37 items (26 neurological evaluation, 8 MD and 3 behaviour).	Preterm and full-term	2–24 months CA	5–15 min	Direct observation. 0–3 pts each item. Write down asymmetries.	TS, optimal or non-optimal, and motor prognosis.	Intra-R (ICCs 0.98 CI 95%) Inter-R (ICCs 0.98 CI 95%)	94.4%
PDMS-2, M. Rhonda Folio and Rebecca R. Fewell, 2000 [23]	USA	Observational evaluation (gross and fine MD). 249 items (8 MR, 30 postural control, 89 locomotion, 24 object handling, 26 grip and 72 VC).	Children with MD delay	0–5 years	45–60 min	Direct observation. It is initiated at any age between 0 and 5 years. 0–2 pts. It is terminated when three consecutive zeros are obtained.	SS, percentile and DQ. Risk of motor delay.	Inter-R (ICCs 0.758–0.999 CI 95%) IC (α 0.991–0.999)	100%
GMFM-88. Dianne J. Russell, Peter L. Rosenbaum, Marilyn Wright, and Lisa M. Avery, 1989 [24,25]	UK	Observational scale. 88 items (17 lying down and turning over, 20 St, 14 crawling and kneeling, 13 S, 24 walking, running and jumping).	Children with CP	5 months- 16 years	45–60 min	Direct observation. 0–3 pts. Write down the use of walking devices.	Total percentage of MD and each dimension.	Inter-R (ICCs 0.99–1.00, CI 95%) Intra-R (ICCs 0.99–1.00, CI 95%)	77.8%
NBAS, T. Berry Brazelton, 1973 [26]	USA	Neonatal behaviour scale. 53 items (18 autonomous behaviour, 18 MD, 7 self-regulation, 10 social behaviour).	TD or preterm babies	0–2 months	30–45 min	Direct observation in Sp or semi-seated. 0–9 pts.	TS (profile) expected behaviour or risk.	IC (α 0.65)	16.7%
Premie-Neuro, Diane K. Daily and Patricia H. Ellison, 2005 [27]	USA	Neuromotor assessment. 24 items (8 neurological, 8 movement and 8 response capacity).	Preterm children (23–37 GW)	0–6 months CA	4–8 min	Direct observation (monitoring device). Hands in the incubator, movements/handling are triggered.	TS: neurologically normal, doubtful, lower than normal.	IC (α 0.72) Inter-R (ICCs 0.524–0.901 CI 95%)	38.9%
TIMP, Suzann K. Campbell, Gay L. Girolami y Thubi HA Kolobe, Elizabeth T. Osten and Maureen C. Lenke, 1900 [28]	USA	Early MD scale. 42 items (13 MB, 29 MR).	Premature and full-term babies	34 GW–4 months CA	20–30 min	Direct observation and triggered responses. 0–6 pts (MB) and 0–3 pts (MR).	TS: risk of neuromotor alteration.	IC (α > 0.95) Intra-R (ICCs 0.994 CI 95%).	27.8%

TD: typical development, TS: total score, PP: prone position, SP: supine position, Si: sitting, St: standing, MD: motor development, DQ: developmental quotient, GW: gestational week, CA: corrected age, IC: internal consistency, inter-R: interobserver reliability, intra-R: intraobserver reliability, ICCs: intraclass correlation coefficients, CI: confidence interval, CoV: Concurrent validity, VC: visuomotor coordination, SS: scaled score, MB: motor behaviour, MR: motor responses, CP: cerebral palsy.

**Table 2:** Subdomain of the included scales (characteristics of the motor development measure).

Scales	Gross Motor Development					Reflexes	Postural Control	Balance		Coordination		Locomotion	Object Handling	Grip	Muscle tone	Body Alignment: Asymmetries	Social Behaviour and Reactions to Stimuli	Movements (Quality, Fluidity, Symmetry)
	PP	SP	Si	St	W			Static	Dynamic	Body	Eye-hand							
1. AIMS [22]	✔	✔	✔	✔	✔		✔											
2. HINE [8]	✔	✔	✔	✔	✔	✔	✔							✔	✔	✔	✔	✔
3. PDMS-2 [23]	✔	✔	✔	✔	✔	✔	✔	✔	✔	✔	✔	✔	✔	✔				
4. GMFM-88 [24,25]	✔	✔	✔	✔	✔		✔											
5. NBAS [26]						✔	✔								✔		✔	✔quality
6. Premie-Neuro [27]						✔	✔								✔	✔	✔	✔symmetry
7. TIMP [28]						✔	✔									✔	✔	✔symmetry

SP: supine position; PP: prone position; Si: sitting; St: standing; W: walking; AIMS: Alberta Infant Motor Scale; HINE: Hammersmith Infant Neurological Examination; PDMS-2: Peabody Motor Developmental Scale; GMFM: Gross Motor Function Measure; NBAS: Neonatal Behavioural Assessment Scale; TIMP: Test of Infant Motor Performance.

**Table 3:** Evaluation of the methodological quality with QUADAS-2.

Studies	Risk of Bias				Applicability Results			
	Patient Selection	Index Test	Reference Test	Flow and Times		Patient Selection	Index Test	
AIMS, Martha C. Piper and Johanna Darra, 1994 [22]	?	☺	?	?		☺	☺	?
HINE, Lilly Dubowitz, 1981 [8]	?	☺	?	☺		☺	☺	?
PDMS-2, M. Rhonda Folio and Rebecca R. Fewell, 2000 [23]	?	☺	☹	☺		☺	☺	?
GMFM-88. Dianne J. Russell, Peter L. Rosenbaum, Marilyn Wright, and Lisa M. Avery, 1989 [24,25]	?	☺	?	☺		☹	☺	?
NBAS, T. Berry Brazelton, 1973 [26]	☺	☹	?	☹		☺	?	?
Premie-Neuro, Diane K. Daily and Patricia H. Ellison, 2005 [27]	?	☺	?	☺		☹	☺	?
TIMP, Suzann K. Campbell, Gay L. Girolami y Thubi HA Kolobe, Elizabeth T. Osten and Maureen C. Lenke, 1900 [28]	?	☺	?	?		☺	☺	?

☺; Low probability. ☹ High probability. ? Uncertain probability.

**Table 4:** Evaluation of the methodological quality with the COSMIN verification list.

Studies	Internal Consistency	Reliability	Content Validity	Structural Validity	Criterion Validity	Sensitivity	Transcultural Validity
AIMS, Martha C. Piper and Johanna Darra, 1994 [22]	+++	+++	+++	+++	++	++	
HINE, Lilly Dubowitz, 1981 [8]	++	++	+++	+++	++	+++	
PDMS-2, M. Rhonda Folio and Rebecca R. Fewell, 2000 [23]	+++	+++	++	+++	++	+++	
GMFM-88. Dianne J. Russell, Peter L. Rosenbaum, Marilyn Wright, and Lisa M. Avery, 1989 [24,25]	++	+++	+++	++	++	++	+++
NBAS, T. Berry Brazelton, 1973 [26]	++	+	+++	++	+	++	
Premie-Neuro, Diane K. Daily and Patricia H. Ellison, 2005 [27]	++	++	++	++	++	++	
TIMP, Suzann K. Campbell, Gay L. Girolami y Thubi HA Kolobe, Elizabeth T. Osten and Maureen C. Lenke, 1900 [28]	+++	+++	+++	+++	++	+++	

+ inadequate; ++ doubtful; +++ adequate; ++++ very good.
